# Examining differences in exposure to digital marketing of unhealthy foods reported by Canadian children and adolescents in two policy environments

**DOI:** 10.1186/s40795-025-01019-z

**Published:** 2025-02-07

**Authors:** Laura Vergeer, Carolina Soto, Mariangela Bagnato, Elise Pauzé, Ashley Amson, Tim Ramsay, Dana Lee Olstad, Vivian Welch, Monique Potvin Kent

**Affiliations:** 1 School of Epidemiology and Public Health, Faculty of Medicine, University of Ottawa, Ottawa, ON Canada; 2 Interdisciplinary School of Health Sciences, University of Ottawa, Ottawa, ON Canada; 3 Ottawa Hospital Research Institute, Ottawa Hospital, Ottawa, ON Canada; 4 Department of Community Health Sciences, Cumming School of Medicine, University of Calgary, Calgary, AB Canada; 5 Bruyère Research Institute, Ottawa, ON Canada

**Keywords:** Food marketing, Children, Adolescents, Digital marketing, Unhealthy food

## Abstract

**Background:**

There has been relatively little research on youth’s exposure to food marketing on digital media, which is important as new digital platforms emerge and youth spend more time online. Evidence evaluating different policy approaches to restricting digital food marketing to children is also limited. This study examined differences in self-reported exposure to digital food marketing between children and adolescents in different policy environments: Ontario (where food marketing is self-regulated) and Quebec (where advertising is government regulated).

**Methods:**

An observational cross-sectional online survey was conducted in April 2023 among children (aged 10–12 years) and adolescents (13–17 years) from Ontario and Quebec, recruited by Leger Marketing. Participants self-reported their frequency of exposure to food marketing via various digital platforms and marketing techniques. Logistic regression and proportional odds models examined differences in exposure by province and age group, adjusting for sociodemographic characteristics and digital device usage.

**Results:**

The odds of reporting more frequent exposure to marketing of sugary drinks (OR: 0.48; 95% CI: 0.33, 0.69), sugary cereals (OR: 0.59; 95% CI: 0.41, 0.86), salty/savoury snacks (OR: 0.67; 95% CI: 0.47, 0.96), fast food (OR: 0.65; 95% CI: 0.45, 0.92), and desserts/sweet treats (OR: 0.54; 95% CI: 0.37, 0.78) were lower among Quebec children than Ontario children. Quebec children were less likely than Ontario children (OR: 0.56; 95% CI: 0.38, 0.84), but more likely than Quebec adolescents (OR: 1.58; 95% CI: 1.04, 2.42), to report exposure to unhealthy food marketing on one or more gaming/TV/music streaming platform/website(s). Compared with Ontario children, Quebec children were less likely to report exposure to marketing featuring characters or child/teenage actors (OR: 0.51; 95% CI: 0.34, 0.76), child-appealing subjects, themes and language (OR: 0.59; 95% CI: 0.40, 0.89), and visual design, audio and special effects (OR: 0.64; 95% CI: 0.41, 0.99), and to report exposure to a greater number of marketing techniques (OR: 0.60; 95% CI: 0.43, 0.84).

**Conclusions:**

Exposure to unhealthy food marketing on digital media is high for youth from Ontario and Quebec, particularly for Ontario children. These findings reinforce the need for federal regulations to protect Canadian youth from unhealthy food marketing on digital media.

**Supplementary Information:**

The online version contains supplementary material available at 10.1186/s40795-025-01019-z.

## Background

Unhealthy dietary patterns are a leading risk factor in the development of obesity and other diet-related non-communicable diseases in Canada and globally [1]. Overall, the diet quality of Canadian children and adolescents (hereafter referred to as “youth”) is poor, characterized by inadequate intakes of fruits and vegetables and overconsumption of highly processed, energy-dense foods and beverages (hereafter referred to as “foods”) that are high in sodium, saturated fat and/or free sugars [2, 3]. Diet quality is greatly influenced by the environments in which people make food choices, including how food is marketed [4]. Food marketing can have detrimental impacts on youth’s nutrition knowledge, food preferences, food purchases (or purchase requests), and dietary patterns [5]. Youth are especially vulnerable to the impacts of food marketing given their underdeveloped cognitive skills, susceptibility to peer influence and frequent marketing exposure [6, 7]. Moreover, the products marketed to youth are typically of low nutritional quality, contributing to overconsumption and high intakes of nutrients of public health concern [5]. Frequent consumption of unhealthy foods influenced by exposure to powerful marketing of such foods increases youth’s risk of developing overweight or obesity and other chronic conditions, such as high blood pressure, type 2 diabetes, cardiovascular disease and dental decay [8, 9]. Unhealthy food marketing to youth has therefore been identified as a public health concern in Canada and elsewhere [5, 8, 10–12].

Unhealthy food marketing has historically been, and continues to be prevalent on food packaging, television, and in settings where youth gather (e.g., school, sports clubs) [13, 14]. There is, however, growing concern over youth’s exposure to unhealthy food marketing in digital spaces (e.g., social media, websites, gaming platforms), particularly as they spend an increasing amount of time online [15]. Findings from a recent study indicated that 87% of Canadians aged 12–17 years reported owning a smartphone in the previous month, as did 49% of those aged 7–11 years [16]. Digital marketing facilitates user engagement, amplifying the marketing message and its impact as youth add ‘likes’ or ‘comments’ to posts and ‘share’ them with friends and family [10]. Moreover, data on users’ online activities and preferences can be used by food companies and websites or social media platforms to personalize and target marketing [17]. The growth of digital marketing of food has also introduced a suite of novel marketing techniques (e.g., influencer marketing), thereby increasing marketing exposure, frequency and persuasive power [5].

Digital food marketing to youth has been relatively underexamined. Most research in this area has focused on food marketing to youth via more traditional forms of digital media, such as websites, with very few studies on social media exposure [13, 18]. Additionally, few studies have examined food marketing by social media influencers or on gaming platforms, despite them becoming increasingly popular among children and adolescents [19]. Research conducted in Canada shows that unhealthy food marketing is highly prevalent on children and adolescents’ preferred websites, and that youth are frequently exposed to it on social media [18, 20–25]. For example, one study identified 54 million food advertisements over a one-year period on the 10 websites most popular with Canadian children, with restaurant foods and cakes, cookies, and ice cream being advertised most frequently [22]. Another study in Canada found that 72% of participating children and adolescents were exposed to food marketing on social media over a 10-minute period, with fast food and sugar sweetened beverages being the most promoted [20]. There is, however, a need for additional research to characterize youth’s exposure to unhealthy food marketing on a broader array of digital platforms where they spend significant amounts of time (e.g., TV streaming and gaming platforms, social media).

The World Health Organization (WHO) and other public health authorities have called on countries to introduce regulations to protect youth from unhealthy food marketing, including in digital spaces [5]. Throughout most of Canada, food marketing is self-regulated by the food industry through the *Code for the Responsible Advertising of Food and Beverage Products to Children* (CCFBA; implemented in June 2023 and preceded by the Canadian Children’s Food and Beverage Advertising Initiative (CAI)). Companies can voluntarily commit to only advertising foods that meet the Code’s nutrition criteria in a manner that does not explicitly target children on certain media primarily directed to children under the age of 13 [26, 27]. However, studies conducted in Canada and globally have shown that voluntary policies are largely ineffective in limiting children’s exposure to unhealthy food marketing and may increase marketing exposure and power [5, 28]. In Canada, only the province of Quebec has a mandatory policy that limits child-directed commercial advertising, including food advertising. The province’s Consumer Protection Act restricts all commercial advertising to children under 13 years of age [29]. Since 2016, legislation and regulations have been proposed at the federal level to restrict unhealthy food marketing to children under 13; however, they have yet to be adopted [8, 30]. Irrespective of whether these regulations are put into effect, Canadian adolescents aged 13 years and older will remain unprotected from unhealthy food marketing.

Overall, there is relatively little research on exposure to digital food marketing and the marketing strategies employed on digital media among children and adolescents in Canada and globally. There is also limited research on differences in exposure between age groups and policy environments, which is a significant knowledge gap. Moreover, while some studies have examined Ontario children’s exposure to unhealthy food marketing on digital media, no such evaluations have been conducted for children in Quebec, where children under age 13 are protected from commercial advertising through legislation. The purpose of this study was to examine differences between provinces (Ontario versus Quebec) and age groups (children aged 10–12 years versus adolescents aged 13–17 years) in self-reported frequency of exposure to food marketing via multiple digital platforms and marketing techniques. Given Quebec’s Consumer Protection Act and the lack of food marketing regulations in Ontario, it was hypothesized that children from Quebec would report less exposure to digital marketing of unhealthy foods than Quebec adolescents or Ontario children.

## Methods

### Study protocol

This study used data from an observational cross-sectional survey that was conducted in April 2023 among youth aged 10–17 years in Ontario and Quebec. The survey aimed to examine youth’s self-reported exposure to food marketing across all social media platforms (e.g., YouTube, Instagram, TikTok) and other types of digital media channels (e.g., gaming, TV and music streaming platforms). Survey questions were adapted from the International Food Policy Study Youth Survey [31]; the survey used in this study is provided in **Additional File 1.** The sample was stratified by sex (600 males and 600 females), by policy environment (Quebec and Ontario) and by age (10–12 years and 13–17 years). The sample size was selected to enable estimation of mean responses within +/- 16% of one standard deviation within each sex/region/age stratum and provide 80% power to detect a small-to-moderate effect (Cohen’s d) of 0.33 between sex/region/age strata. Participants were recruited by Leger Marketing [32], a market research company, to complete a self-administered online survey. Ontario and Quebec were selected for recruitment because they are the most populous provinces in Canada and they differ in how they regulate advertising to children under age 13. Children and adolescents aged 10–17 years were selected because they frequently use social media, are understudied and can complete surveys independently without parental assistance. Additionally, Quebec children aged 10–12 years are targeted by the province’s Consumer Protection Act [29], while those older than 13 years are not targeted by any marketing regulations (in either province), thereby serving as a comparison group.

Leger survey panelists who identified as parents were sent an email invitation to participate in the survey. Panelists were eligible to participate if they lived in Ontario or Quebec, and had a child between the ages of 10 and 17 years with access to a digital device that could be used to complete an online survey. Parent participants completed a short survey about sociodemographic characteristics of their child and household. Youth participants completed a separate survey to provide information about their age, sex, usual digital device use (amount of time spent online and platforms or websites most frequently accessed) and ownership, self-reported exposure to unhealthy food advertising across various digital media sources in the previous week and to related digital marketing techniques. Participants who completed the survey received either cash or virtual incentives redeemable for gift cards as compensation. This study was approved by the University of Ottawa Research Ethics Board (file H-11-21-7343). Both parent/guardian and youth participants completed consent and assent forms, respectively, prior to participating.

### Measures

#### Self-reported exposure to digital food marketing

Youth respondents were asked to report the frequency at which they had been exposed to advertisements for each of the following foods while using their smartphone, tablet, laptop or desktop computer in the previous 7 days: sugary drinks (e.g., soda/pop, sports drinks, energy drinks, juices); sugary cereals, fruits and/or vegetables; salty/savoury snacks (e.g., potato chips, pretzels, cheese puffs); fast food (e.g., pizza, French fries, burgers); and desserts or treats (e.g., cookies, ice cream, candy). Response options included: “never”; “1–3 times during the week”; “4–6 times during the week”; “every day”; “more than once a day”; and “prefer not to answer”.

Participants were also asked to indicate whether they recalled seeing or hearing advertisements for ‘unhealthy’ food or drinks in the previous 7 days on specific social media platforms (including Facebook, Instagram, X (formerly known as Twitter), TikTok, Snapchat, YouTube, Pinterest, blogs/websites, posts and videos shared by influencers, and posts and videos shared by friends on social media) and/or gaming, TV or music streaming platforms/websites, which included Twitch, gaming websites (e.g., Roblox), livestreamed games or eSports, TV streaming platforms (e.g., Netflix, Prime Video), and Spotify. Response options included “yes”, “no”, “I don’t use this platform” and “prefer not to answer”. Variables were recoded into a binary format by combining the “no” and “I don’t use this platform” response options (and coding responses of “prefer not to answer” as missing).

Self-reported exposure to digital marketing techniques was assessed by asking participants to report whether, in the previous 7 days, they had been exposed to digital marketing of unhealthy food that used: characters or child/teenage actors (including cartoon characters from movies or TV, characters owned by food companies, and child or teenage actors); child-appealing subjects, themes and language (including slang used by children or teenagers, themes like magic, mystery, adventure or heroes, as well as themes like fun, popularity, being cool, being fashionable, being free and independent); celebrities, public figures and sports (including celebrities from movies/TV/bands, social media influencers, athletes or favourite sports teams, and extreme sports); child-appealing visual design, audio and special effects (including appealing bright colours, catchy/popular music, and special effects/animation); references to product benefits, health or nutrition (including highlighting new products, saying the product is convenient, and promoting its health or nutrition benefits); incentives and premiums (including contests, free giveaways, limited time offers, price promotions, and reward/incentive programs); cross-promotions (including a link to an event, movie or TV show); and engagement techniques (including activities/polls/games/quizzes, and encouragement to like, comment or share content with friends on social media). Participants reported exposure to each of the 25 marketing techniques examined; there was an option to select “prefer not to answer” in response to each question. Similar techniques were subsequently pooled together for analytical purposes; these categories were based on those used in Health Canada’s 2023 policy proposal for restrictions on food marketing to children [8].

#### Use of digital devices

Youth participants were asked to report whether they owned a smartphone and/or a tablet and/or a laptop or desktop computer, or whether they had access to family members’ devices. From these variables, it was determined how many of these device types were owned by participants: none; one; two; or all three (i.e., owns a smartphone, a tablet and a laptop or desktop computer). Participants also reported, for both weekdays and weekend days, their usual time spent on various online activities, including: watching YouTube; on social media (including messaging, posting, or liking posts on platforms such as Instagram, X, TikTok, Snapchat, Facebook); watching TV shows or movies on television streaming platforms (e.g., Disney+, Netflix); playing games on smartphones, computers or game consoles; browsing online, reading websites, Googling, etc.; and watching gaming or livestreaming content on Twitch. Responses were captured using a scale (0 h, up to 15 min, up to 30 min, up to 1 h, up to 2 h, up to 3 h, up to 4 h, more than 4 h, or prefer not to answer). To derive an estimate of total screen time and average screen time by media channel/activity, a similar approach to Demers-Potvin et al. (2022) [33] was adopted. Responses were converted into minutes, using the ceiling response value, such that ‘up to 1 hour’ was recoded as 60 min, ‘more than 5 hours’ was recoded as 300 minutes (5 hours). ‘Prefer not to answer’ was recoded as missing data. The sum of exposure across all media types (i.e., total number of minutes spent online) was generated for weekdays and weekend days. Winsorization was used to identify and limit the effect of extreme values. Since the distributions of weekend and weekday total screen times were highly skewed, a non-parametric Winsorization approach was used, whereby the top 5% of values were reduced to the 95th percentiles (1080 minutes for weekdays and 1140 minutes for weekend days). The Winsorized values were then summed to generate a weighted estimate for total screen time per day (with total weekday and weekend day screen times weighted as 5/7 and 2/7, respectively).

#### Sociodemographic characteristics

The survey collected information about youth’s sociodemographic characteristics, including age (in years), sex at birth (male or female), province (Ontario or Quebec), race, height and weight, and annual household income level. Participants aged 10–12 years and 13–17 years will hereafter be referred to as children and adolescents, respectively. Race was determined by asking the parents: “Which race category(ies) best describes your child? Check all that apply.” Response options included Black, East Asian, South Asian, Southeast Asian, Indigenous, Latin American, Middle Eastern, White, ‘other race category’ and ‘prefer not to answer’. Participants who selected more than one race were recoded into an “other or mixed” race category. Due to the relatively small numbers of observations in the minority race categories, responses were collapsed into “White” and “Other”, with the latter category encompassing all responses except “White” and “prefer not to answer”. BMI was calculated using youth’s heights and weights as reported by their parents and classified according to the WHO’s BMI-for-age cut-offs for youth as having “severe thinness”, “thinness”, “normal weight”, “overweight” or “obesity” [34]. Extreme BMI values were classified as Z-scores > 4 or <-4, as the WHO BMI-for-age cut-offs do not extend beyond 4 standard deviations. Responses of “severe thinness” and “thinness” were combined due to low numbers of observations in these categories. Lastly, annual household income level was determined by asking parents to estimate their total annual household income from all sources, before taxes and deductions, during 2022. Responses (in CAD) were collapsed into “less than $50,000”, “$50,000 to less than $100,000”, “$100,000 to less than $150,000”, “over $150,000”, and “prefer not to answer”.

### Analyses

The survey was completed by 1211 participants. Those who selected “prefer not to answer” for any of the variables concerning sociodemographic characteristics, digital device ownership, time spent online and/or self-reported exposure to digital food marketing were excluded from this analysis (*n* = 243), resulting in an analytic sample size of 968 children (*n* = 481) and adolescents (*n* = 487). Given the large number of participants with missing or extreme height/weight measures or BMI values (*n* = 258), a separate “extreme values/missing” category was created and kept in the sample, as has been done in comparable previous research [33, 35–40].

Descriptive statistics were tabulated for the sociodemographic, digital device ownership, time spent online, and digital marketing exposure variables, presented by province and age group, and for the total sample. For the digital platform (social media and gaming/TV/music streaming platforms/websites) and marketing technique categories (e.g., characters or child/teenage actors; subjects, themes and language), descriptive statistics reflect the number and proportion of participants who reported exposure to one or more platforms or techniques in that category. To examine differences in the frequency of digital marketing exposure by province and age group, separate proportional odds logistic regression models were constructed for each type of food advertisement (sugary drinks, sugary cereals, salty or savoury snacks, fruits or vegetables, fast food, and desserts or sweet treats). For these analyses, the response options indicating exposure “every day” and “more than once a day” were combined due to relatively small numbers of observations (particularly in the latter group). Models included an interaction term between province and age group, and were adjusted for sex, annual household income, race, BMI, electronic device ownership and usual amount of time spent online. Electronic device ownership was included since there are differences in electronic device usage and ownership between children and adolescents in Canada [20], and marketing uses digital footprints and user profiles to personalize and target marketing, which may affect the frequency and power of marketing for children owning one or more devices [41]. Similarly, models were adjusted for online screen time due to differences between age groups [16], and screen time has been shown to be positively associated with food marketing exposure [33]. Separate binary logistic regression models were used to examine differences between provinces and between age groups in terms of exposure to marketing on digital platforms (i.e., social media; gaming, TV or music streaming platforms/websites), as well as exposure to various types of marketing techniques (e.g., characters or child/teenage actors; subjects, themes and language) using an interaction term between province and age group, and with adjustment for sex, annual household income, race, BMI, electronic device ownership, and time spent online. A proportional odds model was also used to examine differences in the number of marketing techniques (up to 25) to which participants were exposed, using the same age*province interaction term and covariates as the other models. Analyses were conducted using IBM SPSS Statistics (Version 29.0.1.0), and statistical significance was set at *p* < 0.05 for all models.

## Results

### Sociodemographic characteristics and digital device usage

Of the 968 participants included in the analytic sample, approximately half were from Ontario (50.4%), aged 10–12 years (49.7%) and/or male (51.2%; Table 1). Youth were predominately White (75.2%), particularly in Quebec, where 90.4% children and 91.3% of adolescents identified as White. Additionally, nearly half of participants were of normal weight (46.6%), and 37.9% were from households with reported incomes ranging from $50K to less than $100K. Most of the sample (92.4%) owned at least one type of electronic device (smartphone, tablet and/or laptop or desktop computer), although there were some differences between provinces and age groups. For example, 18.8% of children from Quebec reported not owning a smartphone, tablet or computer, compared with only 2.1% of adolescents in that province and 7.9% of children from Ontario. The mean ± SD amount of time spent online per day was 436.6 ± 264.8 min for the overall sample and was highest for adolescents in Quebec (489.9 ± 288.1 min). Among the overall sample, mean amount of time spent online was highest for YouTube on both weekdays (90.6 ± 80.5 min) and weekend days (113.9 ± 84.9 min).

**Table 1:** Sociodemographic characteristics and use of electronic devices among participating children (10–12 years) and adolescents (13–17 years) (*n* = 968)

	Ontario		Quebec		Total sample (*n* = 968)
	Children (*n* = 242)	Adolescents (*n* = 246)	Children (*n* = 239)	Adolescents (*n* = 241)	
	*n* (%)	*n* (%)	*n* (%)	*n* (%)	*n* (%)
Sex					
Male	126 (52.1)	127 (51.6)	122 (51.0)	121 (50.2)	496 (51.2)
Female	116 (47.9)	119 (48.4)	117 (49.0)	120 (49.8)	472 (48.8)
Race					
White	155 (64.0)	137 (55.7)	216 (90.4)	220 (91.3)	728 (75.2)
Other	87 (36.0)	109 (44.3)	23 (9.6)	21 (8.7)	240 (24.8)
BMI classification					
Severe thinness or thinness	3 (1.2)	10 (4.1)	7 (2.9)	11 (4.6)	31 (3.2)
Normal weight	100 (41.3)	134 (54.5)	87 (36.4)	130 (53.9)	451 (46.6)
Overweight	40 (16.5)	36 (14.6)	37 (15.5)	36 (14.9)	149 (15.4)
Obesity	30 (12.4)	16 (6.5)	18 (7.5)	15 (6.2)	79 (8.2)
Extreme values/not stated	69 (28.5)	50 (20.3)	90 (37.7)	49 (20.3)	258 (26.7)
Annual household income (CAD)					
Less than $50,000	46 (19.0)	34 (13.8)	36 (15.1)	36 (14.9)	152 (15.7)
$50,000 to less than $100,000	96 (39.7)	109 (44.3)	75 (31.4)	87 (36.1)	367 (37.9)
$100,000 to less than $150,000	54 (22.3)	64 (26.0)	71 (29.7)	63 (26.1)	252 (26.0)
Over $150,000	46 (19.0)	39 (15.9)	57 (23.8)	55 (22.8)	197 (20.4)
Computer (laptop or desktop), tablet and smartphone ownership					
Does not own a device	19 (7.9)	5 (2.0)	45 (18.8)	5 (2.1)	74 (7.6)
Owns at least one type of device	53 (21.9)	3.7 (15.0)	105 (43.9)	63 (26.1)	258 (26.7)
Owns at least two types of devices	99 (40.9)	99 (40.2)	69 (28.9)	117 (48.5)	384 (39.7)
Owns all three types of devices	71 (29.3)	105 (42.7)	20 (8.4)	56 (23.2)	252 (26.0)
Mean (SD)					
Time spent online per day, weighted^a^ (minutes)	415.4 (237.6)	481.6 (267.4)	358.0 (242.5)	489.9 (288.1)	436.6 (264.8)
Weekday: Time spent online per day (minutes)					
Watching YouTube	90.5 (70.6)	89.9 (75.0)	91.8 (92.1)	90.2 (83.5)	90.6 (80.5)
On social media	52.2 (62.5)	101.3 (88.2)	58.9 (79.4)	124.9 (101.7)	84.4 (89.2)
Watching TV streaming platforms	83.6 (65.2)	84.1 (68.3)	75.8 (82.3)	101.4 (94.0)	86.2 (78.7)
Playing games	84.2 (74.9)	85.6 (82.9)	80.9 (86.8)	90.8 (98.8)	85.4 (86.2)
Browsing online, reading websites, Googling	38.8 (45.5)	59.6 (62.4)	26.9 (39.0)	59.2 (63.7)	46.2 (55.5)
Watching gaming or livestreaming on Twitch	29.6 (56.8)	36.3 (61.8)	11.0 (29.5)	22.2 (52.1)	24.9 (52.4)
Weekend day: Time spent online per day (minutes)					
Watching YouTube	130.4 (85.6)	119.4 (86.8)	101.2 (82.1)	104.4 (82.1)	113.9 (84.9)
On social media	75.2 (82.2)	121.5 (97.5)	68.1 (84.4)	132.7 (100.9)	99.5 (95.7)
Watching TV streaming platforms	114.0 (82.9)	119.7 (86.7)	93.1 (81.7)	114.7 (88.8)	110.5 (85.6)
Playing games	120.7 (92.0)	114.9 (98.0)	96.5 (91.0)	107.6 (104.7)	110.0 (96.9)
Browsing online, reading websites, Googling	51.7 (57.4)	75.7 (72.7)	25.0 (39.0)	48.9 (53.3)	50.5 (59.7)
Watching gaming or livestreaming on Twitch	38.7 (69.6)	47.7 (73.3)	12.8 (32.4)	24.8 (55.6)	31.1 (61.4)

^a^After non-parametric Winsorization; values were weighted to reflect the proportion of the week consisting of weekdays and weekend days

### Self-reported exposure to digital food marketing

#### Frequency of exposure to digital food marketing

Among the overall sample, self-reported exposure to digital marketing was lowest for fruits and vegetables, with 30.9% of participants indicating they had been exposed to this type of digital marketing in the 7 days preceding the survey (Table 2). Conversely, frequency of exposure to digital marketing was highest for fast food, with 74.3% of participants indicating one or more exposures in the previous week. Compared with Ontario children, a greater proportion of children from Quebec reported having been exposed to digital marketing of sugary drinks (52.7% vs. 79.3%), sugary cereals (42.7% vs. 66.5%), salty or savoury snacks (56.9% vs. 75.6%), fast food (62.3% vs. 82.2%) and desserts or sweet treats (45.2% vs. 66.5%) one or more times in the prior week. Differences between other age groups within or between provinces were less consistent across food categories.

**Table 2:** Youth’s self-reported frequency of exposure to digital food marketing, presented by province and age group (*n* = 968)

Frequency of exposure to marketing of food	Ontario		Quebec		Total (*n* = 968)
	Children (*n* = 242)	Adolescents (*n* = 246)	Children (*n* = 239)	Adolescents (*n* = 241)	
	*n* (%)	*n* (%)	*n* (%)	*n* (%)	*n* (%)
Sugary drinks					
Never	50 (20.7)	69 (28.0)	113 (47.3)	93 (38.6)	325 (33.6)
1–3 times per week	127 (52.5)	103 (41.9)	89 (37.2)	108 (44.8)	427 (44.1)
4–6 times per week	26 (10.7)	21 (8.5)	18 (7.5)	18 (7.5)	83 (8.6)
Every day	26 (10.7)	39 (15.9)	18 (7.5)	16 (6.6)	99 (10.2)
More than once per day	13 (5.4)	14 (5.7)	1 (0.4)	6 (2.5)	34 (3.5)
Sugary cereals					
Never	81 (33.5)	106 (43.1)	137 (57.3)	131 (54.4)	455 (47.0)
1–3 times per week	109 (45.0)	76 (30.9)	68 (28.5)	84 (34.9)	337 (34.8)
4–6 times per week	18 (7.4)	36 (14.6)	19 (7.9)	13 (5.4)	86 (8.9)
Every day	24 (9.9)	21 (8.5)	13 (5.4)	10 (4.1)	68 (7.0)
More than once per day	10 (4.1)	7 (2.8)	2 (0.8)	3 (1.2)	22 (2.3)
Fruits or vegetables					
Never	159 (65.7)	164 (66.7)	169 (70.7)	177 (73.4)	669 (69.1)
1–3 times per week	51 (21.1)	46 (18.7)	45 (18.8)	46 (19.1)	188 (19.4)
4–6 times per week	15 (6.2)	16 (6.5)	14 (5.9)	10 (4.1)	55 (5.7)
Every day	13 (5.4)	15 (6.1)	9 (3.8)	7 (2.9)	44 (4.5)
More than once per day	4 (1.7)	5 (2.0)	2 (0.8)	1 (0.4)	12 (1.2)
Salty or savoury snacks					
Never	59 (24.4)	76 (30.9)	103 (43.1)	86 (35.7)	324 (33.5)
1–3 times per week	108 (44.6)	96 (39.0)	90 (37.7)	101 (41.9)	395 (40.8)
4–6 times per week	32 (13.2)	33 (13.4)	24 (10.0)	30 (12.4)	119 (12.3)
Every day	33 (13.6)	30 (12.2)	20 (8.4)	20 (8.3)	103 (10.6)
More than once per day	10 (4.1)	11 (4.5)	2 (0.8)	4 (1.7)	27 (2.8)
Fast food					
Never	43 (17.8)	50 (20.3)	90 (37.7)	66 (27.4)	249 (25.7)
1–3 times per week	111 (45.9)	86 (35.0)	80 (33.5)	88 (36.5)	365 (37.7)
4–6 times per week	35 (14.5)	50 (20.3)	38 (15.9)	41 (17.0)	164 (16.9)
Every day	39 (16.1)	39 (15.9)	28 (11.7)	37 (15.4)	143 (14.8)
More than once per day	14 (5.8)	21 (8.5)	3 (1.3)	9 (3.7)	47 (4.9)
Desserts or sweet treats					
Never	81 (33.5)	85 (34.6)	131 (54.8)	110 (45.6)	407 (42.0)
1–3 times per week	92 (38.0)	96 (39.0)	77 (32.2)	88 (36.5)	353 (36.5)
4–6 times per week	34 (14.0)	26 (10.6)	11 (4.6)	19 (7.9)	90 (9.3)
Every day	23 (9.5)	29 (11.8)	16 (6.7)	19 (7.9)	87 (9.0)
More than once per day	12 (5.0)	10 (4.1)	4 (1.7)	5 (2.1)	31 (3.2)

The odds of reporting more frequent exposure to marketing of sugary drinks (OR: 0.48; 95% CI: 0.33, 0.69), sugary cereals (OR: 0.59; 95% CI: 0.41, 0.86), salty/savoury snacks (OR: 0.67; 95% CI: 0.47, 0.96), fast food (OR: 0.65; 95% CI: 0.45, 0.92), and desserts/sweet treats (OR: 0.54; 95% CI: 0.37, 0.78) were significantly lower among Quebec children than children from Ontario (Table 3). Similarly, the odds of reporting more frequent exposure to marketing of sugary cereals (OR: 0.69; 95% CI: 0.49, 0.97) and salty/savoury snacks (OR: 0.68, 95% CI: 0.49, 0.96) were lower for Ontario adolescents compared to Ontario children. Ontario adolescents were more likely to report more frequent exposure to marketing of sugary drinks (OR: 1.72; 95% CI: 1.20, 2.47) and sugary cereals (OR: 1.45; 95% CI: 1.01, 2.09) compared to Quebec adolescents.

**Table 3:** Odds ratio estimates from separate proportional odds regression models examining the associations of age group and province with self-reported frequency of exposure to marketing of different types of food (*n* = 968)^a, b^

	χ2, *p*-value	Adjusted OR (95% CI)
Frequency of exposure to marketing of sugary drinks	23.69, *p* < 0.001*	
Quebec children vs. Ontario children		0.48 (0.33, 0.69)*
Ontario adolescents vs. Ontario children		0.83 (0.59, 1.16)
Quebec children vs. Quebec adolescents		1.00 (0.70, 1.45)
Ontario adolescents vs. Quebec adolescents		1.72 (1.20, 2.47)*
Frequency of exposure to marketing of sugary cereals	17.64, *p* < 0.001*	
Quebec children vs. Ontario children		0.59 (0.41, 0.86)*
Ontario adolescents vs. Ontario children		0.69 (0.49, 0.97)*
Quebec children vs. Quebec adolescents		1.25 (0.86, 1.82)
Ontario adolescents vs. Quebec adolescents		1.45 (1.01, 2.09)*
Frequency of exposure to marketing of fruit or vegetables	2.66, *p* = 0.45	
Quebec children vs. Ontario children		1.03 (0.68, 1.56)
Ontario adolescents vs. Ontario children		0.90 (0.62, 1.32)
Quebec children vs. Quebec adolescents		1.37 (0.89, 2.11)
Ontario adolescents vs. Quebec adolescents		1.20 (0.80, 1.83)
Frequency of exposure to marketing of salty or savoury snacks	9.33, *p* = 0.03*	
Quebec children vs. Ontario children		0.67 (0.47, 0.96)*
Ontario adolescents vs. Ontario children		0.68 (0.49, 0.96)*
Quebec children vs. Quebec adolescents		1.09 (0.76, 1.56)
Ontario adolescents vs. Quebec adolescents		1.11 (0.78, 1.59)
Frequency of exposure to marketing of fast food	7.94, *p* = 0.047*	
Quebec children vs. Ontario children		0.65 (0.45, 0.92)*
Ontario adolescents vs. Ontario children		1.03 (0.74, 1.43)
Quebec children vs. Quebec adolescents		0.84 (0.59, 1.19)
Ontario adolescents vs. Quebec adolescents		1.34 (0.94, 1.89)
Frequency of exposure to marketing of desserts or sweet treats	14.28, *p* = 0.003*	
Quebec children vs. Ontario children		0.54 (0.37, 0.78)*
Ontario adolescents vs. Ontario children		0.81 (0.58, 1.14)
Quebec children vs. Quebec adolescents		0.93 (0.65, 1.35)
Ontario adolescents vs. Quebec adolescents		1.41 (0.98, 2.01)

*Indicates statistical significance at alpha = 0.05 level

^a^Models included an interaction term between province and age group, and were adjusted for sex, annual household income, race, BMI, electronic device ownership, and usual amount of time spent online. ^b^The reference category is listed second

#### Platform of exposure

Of the total sample, 71.9% reported exposure to unhealthy food marketing on at least one social media platform in the previous week, with the largest proportion exposed on YouTube (59.2%) (Table 4). Fewer participants were exposed to unhealthy food marketing on one or more gaming, TV or music streaming platforms/websites (39.8%). Moreover, the proportion of Ontario children (78.1%) and adolescents (78.9%) who reported exposure to unhealthy food marketing on at least one social media platform was higher than for children (66.5%) and adolescents (63.9%) from Quebec. Similar results were observed for unhealthy food marketing exposure on gaming, TV or music streaming platforms/websites, which was highest for Ontario children (50.4%), followed by Ontario adolescents (48.8%) and Quebec children (33.1%) and adolescents (26.6%).

**Table 4:** Youth’s self-reported exposure to unhealthy food marketing on various digital platforms (*n* = 968)

Digital platform	Ontario		Quebec		Total (*n* = 968)
	Children (*n* = 242)	Adolescents (*n* = 246)	Children (*n* = 239)	Adolescents (*n* = 241)	
	*n* (%)	*n* (%)	*n* (%)	*n* (%)	*n* (%)
Social media^a^	189 (78.1)	194 (78.9)	159 (66.5)	154 (63.9)	696 (71.9)
Facebook	46 (19.0)	63 (25.6)	37 (15.5)	54 (22.4)	200 (20.7)
Instagram	46 (19.0)	89 (36.2)	24 (10.0)	68 (28.2)	227 (23.5)
X (formerly known as Twitter)	10 (4.1)	39 (15.9)	4 (1.7)	9 (3.7)	62 (6.4)
TikTok	73 (30.2)	103 (41.9)	64 (26.8)	91 (37.8)	331 (34.2)
Snapchat	30 (12.4)	58 (23.6)	18 (7.5)	30 (12.4)	136 (14.0)
YouTube	170 (70.2)	151 (61.4)	141 (59.0)	111 (46.1)	573 (59.2)
Pinterest	18 (7.4)	22 (8.9)	10 (4.2)	14 (5.8)	64 (6.6)
Blogs/websites	38 (15.7)	65 (26.4)	40 (16.7)	37 (15.4)	180 (18.6)
Posts and videos shared by influencers	66 (27.3)	71 (28.9)	48 (20.1)	59 (24.5)	244 (25.2)
Posts and videos shared by friends on social media	48 (19.8)	77 (31.3)	32 (13.4)	49 (20.3)	206 (21.3)
Gaming, TV or music streaming platforms/websites^b^	122 (50.4)	120 (48.8)	79 (33.1)	64 (26.6)	385 (39.8)
Twitch	15 (6.2)	22 (8.9)	7 (2.9)	10 (4.1)	54 (5.6)
Gaming websites (e.g., Roblox)	60 (24.8)	49 (19.9)	40 (16.7)	22 (9.1)	171 (17.7)
Livestreamed games or eSports	22 (9.1)	31 (12.6)	13 (5.4)	16 (6.6)	82 (8.5)
TV streaming platforms (e.g., Netflix, Prime Video)	94 (38.8)	87 (35.4)	40 (16.7)	41 (17.0)	262 (27.1)
Spotify	25 (10.3)	43 (17.5)	16 (6.7)	19 (7.9)	103 (10.6)

^a^The number and proportion of participants who reported being exposed to unhealthy food marketing on at least one social media platform in the 7 days preceding the survey. ^b^The number and proportion of participants who reported being exposed to unhealthy food marketing on at least one gaming, TV or music streaming platform or website in the 7 days preceding the survey

Ontario adolescents were more likely to report exposure to unhealthy food marketing on one or more social media platforms, compared with Quebec adolescents (OR: 1.61; 95% CI: 1.04, 2.49; Table 5). Moreover, Quebec children were less likely to report exposure to unhealthy food marketing on at least one gaming, TV or music streaming platform/website than Ontario children (OR: 0.56; 95% CI: 0.38, 0.84). Children from Quebec (OR: 1.58; 95% CI: 1.04, 2.42) and adolescents from Ontario (OR: 2.36; 95% CI: 1.57, 3.55) were both more likely to report exposure to unhealthy food marketing on one or more gaming, TV or music streaming platforms/websites than Quebec adolescents.

**Table 5:** Odds ratio estimates from logistic regression models examining the associations of age group and province with self-reported exposure to unhealthy food marketing on social media and gaming, TV or music streaming platforms/websites (*n* = 968)^a, b^

Type of digital platform	χ2, *p*-value	Adjusted OR (95% CI)
Social media	9.63, *p* = 0.02*	
Quebec children vs. Ontario children		0.79 (0.51, 1.22)
Ontario adolescents vs. Ontario children		0.85 (0.54, 1.33)
Quebec children vs. Quebec adolescents		1.49 (0.98, 2.26)
Ontario adolescents vs. Quebec adolescents		1.61 (1.04, 2.49)*
Gaming, TV or music streaming platforms/websites	28.32, *p* < 0.001*	
Quebec children vs. Ontario children		0.56 (0.38, 0.84)*
Ontario adolescents vs. Ontario children		0.84 (0.58, 1.21)
Quebec children vs. Quebec adolescents		1.58 (1.04, 2.42)*
Ontario adolescents vs. Quebec adolescents		2.36 (1.57, 3.55)*

*Indicates statistical significance at alpha = 0.05 level

^a^Models included an interaction term between province and age group, and were adjusted for sex, annual household income, race, BMI, electronic device ownership, and usual amount of time spent online. ^b^The reference category is listed second

#### Exposure to digital marketing techniques

More than three-quarters of the total sample (76.7%) reported exposure to one or more marketing techniques (Table 6). Among the overall sample, the greatest proportion of participants reported exposure to one or more techniques related to visual design, audio and special effects (67.4%), with exposure being highest for appealing bright colours (58.3%). Additionally, a greater proportion of Ontario children reported exposure to techniques in the visual design/audio/special effects (77.3%), subjects/themes/language (69.4%), references to product benefits/nutrition/health (65.3%), celebrities/public figures/sports (64.0%), characters or child/teenage actors (62.8%), incentives/premiums (55.4%), cross-promotions (39.7%), and engagement techniques (43.4%) categories, compared with adolescents from the same province, as well as Quebec children and adolescents.

**Table 6:** Youth’s self-reported exposure to unhealthy food marketing using various digital marketing techniques (*n* = 968)

Marketing technique	Ontario		Quebec		Total (*n* = 968)
	Children (*n* = 242)	Adolescents (*n* = 246)	Children (*n* = 239)	Adolescents (*n* = 241)	
	*n* (%)	*n* (%)	*n* (%)	*n* (%)	*n* (%)
Exposed to one or more marketing techniques	203 (83.9)	190 (77.2)	174 (72.8)	175 (72.6)	742 (76.7)
Characters or child/teenage actors^a^	152 (62.8)	130 (52.8)	97 (40.6)	92 (38.2)	471 (48.7)
Cartoon characters from movies or TV	80 (33.1)	68 (27.6)	41 (17.2)	35 (14.5)	224 (23.1)
Characters owned by food companies	107 (44.2)	82 (33.3)	46 (19.2)	56 (23.2)	291 (30.1)
Child or teenage actors	122 (50.4)	93 (37.8)	59 (24.7)	63 (26.1)	337 (34.8)
Subjects, themes and language^a^	168 (69.4)	144 (58.5)	129 (54.0)	132 (54.8)	573 (59.2)
Themes like magic, mystery, adventure or heroes	120 (49.6)	90 (36.6)	62 (25.9)	69 (28.6)	341 (35.2)
Themes like fun, popularity, being cool, being fashionable, being free and independent	143 (59.1)	119 (48.4)	103 (43.1)	108 (44.8)	473 (48.9)
Slang used by children or teenagers	104 (43.0)	98 (39.8)	78 (32.6)	94 (39.0)	374 (38.6)
Celebrities, public figures and sports^a^	155 (64.0)	138 (56.1)	123 (51.5)	129 (53.5)	545 (56.3)
Celebrities from movies, TV or bands	115 (47.5)	96 (39.0)	55 (23.0)	70 (29.0)	336 (34.7)
Social media influencers	95 (39.3)	74 (30.1)	53 (22.2)	67 (27.8)	289 (29.9)
Athletes, favourite sports teams	91 (37.6)	89 (36.2)	73 (30.5)	80 (33.2)	333 (34.4)
Extreme sports	76 (31.4)	72 (29.3)	75 (31.4)	84 (34.9)	307 (31.7)
Visual design, audio and special effects^a^	187 (77.3)	155 (63.0)	158 (66.1)	152 (63.1)	652 (67.4)
Appealing bright colours	172 (71.1)	137 (55.7)	128 (53.6)	127 (52.7)	564 (58.3)
Catchy/popular music	124 (51.2)	108 (43.9)	118 (49.4)	125 (51.9)	475 (49.1)
Special effects/animation	110 (45.5)	83 (33.7)	88 (36.8)	84 (34.9)	365 (37.7)
References to product benefits, health or nutrition^a^	158 (65.3)	140 (56.9)	130 (54.4)	135 (56.0)	563 (58.2)
New products	119 (49.2)	111 (45.1)	106 (44.4)	122 (50.6)	458 (47.3)
Saying the product is convenient	101 (41.7)	83 (33.7)	44 (18.4)	59 (24.5)	287 (29.6)
References to health or nutrition	98 (40.5)	80 (32.5)	65 (27.2)	69 (28.6)	312 (32.2)
Incentives and premiums^a^	134 (55.4)	135 (54.9)	97 (40.6)	113 (46.9)	479 (49.5)
Contests	87 (36.0)	80 (32.5)	49 (20.5)	61 (25.3)	277 (28.6)
Free giveaways	78 (32.2)	80 (32.5)	34 (14.2)	41 (17.0)	233 (24.1)
Limited time offers	84 (34.7)	88 (35.8)	61 (25.5)	68 (28.2)	301 (31.1)
Price promotions	78 (32.2)	83 (33.7)	57 (23.8)	64 (26.6)	282 (29.1)
Reward/incentive programs	69 (28.5)	78 (31.7)	40 (16.7)	50 (20.7)	237 (24.5)
Cross-promotions^a^	96 (39.7)	87 (35.4)	78 (32.6)	78 (32.4)	339 (35.0)
Link to an event	45 (18.6)	49 (19.9)	58 (24.3)	53 (22.0)	205 (21.2)
Link to a movie or TV show	85 (35.1)	76 (30.9)	48 (20.1)	56 (23.2)	265 (27.4)
Engagement techniques^a^	105 (43.4)	90 (36.6)	77 (32.2)	80 (33.2)	352 (36.4)
Activities, polls, quizzes or games	60 (24.8)	59 (24.0)	44 (18.4)	37 (15.4)	200 (20.7)
Encouragement to like, comment or share with your friends on social media	95 (39.3)	75 (30.5)	59 (24.7)	68 (28.2)	297 (30.7)

^a^The number and proportion of participants who reported being exposed to marketing of unhealthy foods featuring one or more of the marketing techniques listed within that category

Compared with Ontario children, Quebec children (OR: 0.51; 95% CI: 0.34, 0.76) and Ontario adolescents (OR: 0.56; 95% CI: 0.39, 0.83) were less likely to report exposure to characters or child/teenage actors (Table 7). Additionally, Ontario adolescents were more likely than adolescents from Quebec to report exposure to techniques in that category (OR: 1.57; 95% CI: 1.06, 2.33). Similarly, Quebec children (OR: 0.59; 95% CI: 0.40, 0.89) and Ontario adolescents (OR: 0.55; 95% CI: 0.37, 0.81) were less likely than Ontario children to report exposure to one or more techniques related to child-appealing subjects, themes and language. Comparable results were also observed for techniques related to visual design, audio and special effects, where exposure was less likely among Quebec children (OR: 0.64; 95% CI: 0.41, 0.99) and Ontario adolescents (OR: 0.45; 95% CI: 0.30, 0.68), compared with children from Ontario. Ontario adolescents were also less likely than children from that province to report exposure to marketing featuring celebrities/public figures/sports (OR: 0.63; 95% CI: 0.43, 0.92) and engagement techniques, such as games, activities or encouragement to share content on social media (OR: 0.67; 95% CI: 0.46, 0.98). Lastly, the odds of being exposed to a greater number of marketing techniques was lower for Quebec children (OR: 0.60; 95% CI: 0.43, 0.84) and Ontario adolescents (OR: 0.62; 95% CI: 0.45, 0.85), compared with children from Ontario (results not shown).

**Table 7:** Odds ratio estimates from separate logistic regression models examining the associations of age group and province with exposure to various types of digital marketing techniques (*n* = 968)^a, b^

Type of marketing technique	χ2, *p*-value	Adjusted OR (95% CI)
Characters or child/teenage actors	27.05, *p* < 0.001*	
Quebec children vs. Ontario children		0.51 (0.34. 0.76)*
Ontario adolescents vs. Ontario children		0.56 (0.39, 0.83)*
Quebec children vs. Quebec adolescents		1.43 (0.96, 2.13)
Ontario adolescents vs. Quebec adolescents		1.57 (1.06, 2.33)*
Subjects, themes and language	15.27, *p* = 0.002*	
Quebec children vs. Ontario children		0.59 (0.40, 0.89)*
Ontario adolescents vs. Ontario children		0.55 (0.37, 0.81)*
Quebec children vs. Quebec adolescents		1.24 (0.84, 1.83)
Ontario adolescents vs. Quebec adolescents		1.14 (0.77, 1.69)
Celebrities, public figures and sports	8.81, *p* = 0.03*	
Quebec children vs. Ontario children		0.69 (0.46, 1.02)
Ontario adolescents vs. Ontario children		0.63 (0.43, 0.92)*
Quebec children vs. Quebec adolescents		1.18 (0.80, 1.74)
Ontario adolescents vs. Quebec adolescents		1.08 (0.73, 1.59)
Visual design, audio and special effects	18.28, *p* < 0.001*	
Quebec children vs. Ontario children		0.64 (0.41, 0.99)*
Ontario adolescents vs. Ontario children		0.45 (0.30, 0.68)*
Quebec children vs. Quebec adolescents		1.40 (0.93, 2.11)
Ontario adolescents vs. Quebec adolescents		0.98 (0.66, 1.47)
References to product benefits, health or nutrition	5.72, *p* = 0.13	
Quebec children vs. Ontario children		0.78 (0.52, 1.16)
Ontario adolescents vs. Ontario children		0.64 (0.44, 0.94)
Quebec children vs. Quebec adolescents		1.10 (0.74, 1.62)
Ontario adolescents vs. Quebec adolescents		0.90 (0.61, 1.33)
Incentives and premiums	4.58, *p* = 0.21	
Quebec children vs. Ontario children		0.67 (0.45, 0.99)
Ontario adolescents vs. Ontario children		0.88 (0.61, 1.28)
Quebec children vs. Quebec adolescents		0.89 (0.60, 1.32)
Ontario adolescents vs. Quebec adolescents		1.18 (0.80, 1.73)
Cross-promotions	2.89, *p* = 0.41	
Quebec children vs. Ontario children		0.80 (0.54, 1.21)
Ontario adolescents vs. Ontario children		0.75 (0.51, 1.10)
Quebec children vs. Quebec adolescents		1.07 (0.71, 1.62)
Ontario adolescents vs. Quebec adolescents		1.0 (0.66, 1.50)
Engagement techniques	7.92, *p* = 0.048*	
Quebec children vs. Ontario children		0.69 (0.46, 1.03)
Ontario adolescents vs. Ontario children		0.67 (0.46, 0.98)*
Quebec children vs. Quebec adolescents		1.16 (0.77, 1.75)
Ontario adolescents vs. Quebec adolescents		1.13 (0.75, 1.69)

*Indicates statistical significance at alpha = 0.05 level

^a^Models included an interaction term between province and age group, and were adjusted for sex, annual household income, race, BMI, electronic device ownership, and usual amount of time spent online. ^b^The reference category is listed second

## Discussion

This study provides a novel comparison of Ontario and Quebec children and adolescents’ self-reported exposure to marketing of several types of foods via a variety of digital platforms and marketing techniques. We found that children and adolescents in Ontario and Quebec reported frequently being exposed to marketing of unhealthy food across numerous digital platforms, using a host of marketing techniques. Exposure was particularly high for Ontario children across food categories, digital platforms and marketing techniques, suggesting the failure of self-regulation in Ontario and the need for government intervention to limit children’s exposure to digital marketing of unhealthy foods. The lower levels of exposure reported by Quebec children (and in some cases, adolescents) compared with those in Ontario suggest that the Quebec Consumer Protection Act may be helping to shield them from unhealthy food marketing on digital media.

Among the total sample, frequency of exposure to digital food marketing was highest for fast food. This is consistent with previous research showing that fast food is frequently promoted to children and adolescents online and in other settings [20, 42, 43]. This finding is also unsurprising given that advertising expenditures are especially high for fast food [44]. Of particular concern is that exposure to fast food marketing is positively associated with brand preferences and intakes of fast food [45], which are typically ultra-processed, energy-dense and high in nutrients of public health concern [46].

In addition, this study found high levels of exposure to unhealthy food marketing on social media (71.9% of the total sample), particularly on YouTube (59.2%), which aligns with studies from other countries that found unhealthy food marketing is prevalent on YouTube and other social media platforms, including on channels popular with children [10, 47, 48]. Like other settings, exposure to food marketing on social media negatively impacts children’s brand and food preferences and intakes [10, 49, 50]. Interestingly, the present study also found exposure to unhealthy food marketing was particularly high on TV streaming platforms (e.g., Netflix, Disney+). To our knowledge, marketing on these platforms has not previously been examined. Given that children and adolescents in this study reported spending upwards of about 1.5–2 h per day on TV streaming platforms, there is a need for future research and policy measures to protect them from exposure to unhealthy food marketing on this medium.

To our knowledge, this is the first study to examine differences between children and adolescents in Ontario and Quebec in terms of their self-reported exposure to marketing of unhealthy foods on social media and TV, gaming and music streaming websites/platforms. Consistent with previous research conducted on television [43, 44, 51], the present study found that although exposure to unhealthy food marketing was high across all age and province groups, it was often greater for children in Ontario than in Quebec. As hypothesized, children from Ontario were more likely to be frequently exposed to marketing of all types of unhealthy foods examined (sugary drinks and cereals, salty snacks, fast food, and desserts/sweet treats), compared with children from Quebec. Ontario children were also more likely than Quebec children to be exposed to marketing on gaming, TV or music streaming platforms/websites, as well as several different types of marketing techniques, and a greater number of marketing techniques overall. These findings suggest that Quebec’s Consumer Protection Act, which extends to marketing on “the web” and “mobile phones”, may be providing some protection to children (under 13 years of age) against unhealthy food marketing on digital media. In addition, compared with Ontario adolescents, those from Quebec were less likely to report frequent exposure to digital marketing of sugary drinks and cereals, unhealthy food marketing on social media and TV/gaming/music streaming platforms, and marketing featuring characters or child/teenage actors, suggesting that the Consumer Protection Act may also be conferring some protection to this age group.

Nonetheless, exposure to unhealthy digital food marketing was high among children in Quebec. For example, more than 60% of Quebec children in this study reported exposure to one or more instances of digital marketing for fast food in the previous week. Similarly, approximately 67% of Quebec children were exposed to digital marketing of unhealthy foods on at least one social media platform, and about 73% were exposed to one or more of the marketing techniques examined. Therefore, there is still room for improvement in the Consumer Protection Act’s ability to protect Quebec children from unhealthy food marketing on digital media. Notably, the most recent guide for the application of the Act as it relates to children’s advertising was published in 2012 [29]. Given the proliferation of new digital media platforms (several of which were examined in this study) [52], the growing number of children owning or accessing electronic devices and the increasing amount of time children are spending online [53, 54], there is a need for an updated guide to outline how these regulations should be applied to digital media platforms, and how compliance will be monitored and enforced.

Findings from this study largely align with those of previous research examining differences between Canadian children and adolescents in online exposure to unhealthy food marketing. Consistent with previous work [20, 42, 55, 56], this research found high levels of exposure to marketing of sugary drinks and cereals, salty/savoury snacks, fast food and desserts/sweet treats among both children and adolescents, and both age groups reported high levels of exposure to several digital marketing techniques. We found almost no difference between children and adolescents in Quebec in their self-reported exposure to digital marketing of unhealthy foods, providing further evidence that the Consumer Protection Act may have similar impacts on both age groups in terms of limiting food marketing exposure. In contrast, Ontario adolescents were less likely than children from that province to be exposed to frequent marketing of sugary drinks and salty/savoury snacks, and were less likely to report exposure to several types of marketing techniques and a larger total number of marketing techniques. The differences in exposure between age groups observed in this study deviate somewhat from the findings of previous research. Acton et al. found 13–17-year-old Canadians were more likely than 10–12-year-olds to report exposure to unhealthy food marketing online [42]. Similarly, Potvin Kent et al. found that, after using their two favourite social media apps for 5 min each, adolescents viewed more instances of food marketing per 10-minute period, on average, than children in Canada (mean [SD] = 2.6 [2.7] versus 1.4 [2.1]) [20]. Differences between studies may be related to methodological heterogeneity. For example, Acton et al. surveyed youth from across Canada (instead of only Ontario and Quebec) and did not examine marketing exposures on individual digital media platforms (survey response options were limited to broad categories such as “website or social media” and “video or computer games”). Moreover, while Potvin Kent et al. estimated children and adolescents’ exposure to food marketing on social media based on their observed usage of two platforms [20], this study investigated exposures across a wider range of digital platforms but relied on self-reported data, which may be subject to misreporting.

In combination, the results of this study illustrate children and adolescents in Ontario and Quebec are frequently exposed to powerful digital marketing of unhealthy foods; however, exposures were particularly high for Ontario children. These findings provide further evidence of the ineffectiveness of voluntary children’s food marketing policies (such as the CAI), and reinforce the urgent need for federal legislation to protect Canadian children from unhealthy food marketing in various settings, including digital media. The Canadian government’s most recent policy proposal (which extends to children under 13 years of age) includes television and digital media and regarding the latter, would be limited to websites, online games, social media or mobile apps that are “rated or described as being specifically intended for children, or where the content is clearly designed for, or to be engaged with by, children” [8]. Given that none of the digital media platforms examined in this study are intended specifically for children and that many digital marketing techniques are used to appeal to audiences beyond children (e.g., celebrities, sports, viral marketing) [10], the proposed policy would likely have limited potential to reduce exposure of children in Ontario (and other provinces) to digital marketing of unhealthy foods.

Overall, this study adds to the global body of evidence demonstrating the need for comprehensive, evidence-based mandatory government restrictions on unhealthy food marketing to children and adolescents, which have been shown to have favourable impacts on youth’s exposure to food marketing and in the use of persuasive marketing techniques [5, 12, 28]. Conversely, industry policies have repeatedly been shown to be ineffective in limiting youth’s exposure to unhealthy food marketing, largely due to narrowness in scope (e.g., in terms of definitions of marketing, age thresholds, media/settings), lax nutrition criteria, and limited adherence due to their voluntary nature [5, 24, 28]. Stringent government policies focused on protecting children’s health rather than profits can limit youth’s exposure to unhealthy food marketing and the detrimental behavioural and health impacts of marketing on this vulnerable population segment [12]. The differences in exposure to digital marketing observed between policy environments and age groups in this study provide further evidence of the ineffectiveness of voluntary industry policies compared with government regulations, and may inform federal policy initiatives to restrict food marketing to youth in Canada and elsewhere. Furthermore, the high levels of digital food marketing exposure observed among Ontario and Quebec youth - particularly for children not protected by government restrictions - reinforce the importance that food marketing policies in Canada and other countries align with WHO recommendations in that they are mandatory, protect children of all ages (i.e., both children and adolescents), are comprehensive across media (including all digital platforms used by youth) and restrict the use of powerful marketing techniques [5]. While the Canadian government’s proposed restrictions on food marketing are strengthened by their mandatory nature, they could be improved by extending to all youth up to 18 years of age and to media/settings beyond television and digital, as recommended by the WHO [5].

### Strengths and limitations

This study provides the first comprehensive comparison of exposure to marketing of different food categories across several digital platforms and examines the variety of marketing techniques between children and adolescents in Ontario and Quebec. This study also accounted for important variables related to sociodemographic characteristics and digital media that may be related to marketing exposure (e.g., sex, income, race, device ownership, time spent online). However, this study is not without limitations. First, this research relied on self-reported exposures to food marketing, which may be subject to measurement error. Moreover, our sample cannot be considered representative, given that the sampling strategy aimed to ensure approximately equal numbers of participants in each province (Ontario, Quebec), age (10–12 years, 13–17 years) and sex (male, female) group. This research was also limited to selected digital media platforms and therefore does not capture all social media and streaming platforms used by children and adolescents. Furthermore, this work did not account for the fact that children may be less likely to recognize marketing than teens, particularly on digital media where the line between advertising and entertainment is more blurred than in other media (e.g., television) [15]. This study also did not examine the number of times youth saw marketing on each digital platform and featuring each marketing technique, thereby potentially underestimating their overall exposure. It is also worth noting that this research only assessed exposure to digital food marketing; it did not examine exposure to marketing via other media/settings, nor did it evaluate the impacts of food marketing on behavioural or nutritional outcomes (e.g., food purchases and intakes). In addition, this analysis did not adjust for multiple comparisons, and the exclusion of 243 participants (20.1%) from the study due to missing data for key variables may have introduced bias. There may also be residual confounding, as this was a cross-sectional study conducted at a single point in time and so no causal relationships can be discerned. Lastly, participants of survey panels like Leger are most often White and of higher socioeconomic status [57], such as in our study, with 75.2% identifying as White and 46.4% reporting annual household incomes of at least $100,000 CAD; this may limit the generalizability of our results.

## Conclusions

Overall, this study found high levels of self-reported exposure to digital marketing of unhealthy foods among children and adolescents in Ontario and Quebec. Exposures were particularly high for Ontario children, reinforcing the need for federal regulations to protect this vulnerable group from unhealthy food marketing on digital media and elsewhere. Rather than limiting their proposed policy to digital media platforms and content rated or described as being specifically for children under 13 years, the Canadian government should consider broadening the scope of their regulations to extend to adolescents (under 18 years of age) and to encompass all digital platforms where youth are present, irrespective of the intended audience. Findings from this research also identify a need for the Quebec government to strengthen their application and monitoring of the Consumer Protection Act to reduce children’s exposure to unhealthy food marketing on digital media, which remains high despite the regulations being in place. Future studies will be warranted to continue monitoring Canadian children and adolescents’ exposure to unhealthy food marketing on digital media, and to monitor policy impacts over time.

## Electronic supplementary material

Below is the link to the electronic supplementary material.

Supplementary Material 1

## Data Availability

Data generated or analyzed during this study are available from the corresponding author upon reasonable request.

## Abbreviations

BMI: Body Mass Index
CAI: Canadian Children’s Food and Beverage Advertising Initiative
CCFBA: Code for the Responsible Advertising of Food and Beverage Products to Children
WHO: World Health Organization
