# Supplementary material for: Examining differences in exposure to digital marketing of unhealthy foods reported by Canadian children and adolescents in two policy environments
Source: BMC Nutr. 2025 Feb 7;11:32. doi: 10.1186/s40795-025-01019-z (PMC11806838; doi:10.1186/s40795-025-01019-z)
Supplement: Supplementary file 1 — Supplementary Material 1 [file 40795_2025_1019_MOESM1_ESM.pdf]

ADDITIONAL FILE 1

TITLE: Examining differences in self-reported exposure to digital marketing of unhealthy foods by Canadian children and adolescents in two policy environments

AUTHORS: Laura Vergeer[1], Carolina Soto[1], Mariangela Bagnato[1], Elise Pauzé[2], Ashley Amson[2], Tim Ramsay[3], Dana Lee Olstad[4], Vivian Welch[1,5], Monique Potvin Kent[1]

AFFILIATIONS:

[1]School of Epidemiology and Public Health, Faculty of Medicine, University of Ottawa, Ottawa, ON, Canada

[2]Interdisciplinary School of Health Sciences, University of Ottawa, Ottawa, Ontario, Canada

[3]Ottawa Hospital Research Institute, Ottawa Hospital, Ottawa, ON, Canada

[4]Department of Community Health Sciences, Cumming School of Medicine, University of Calgary, Alberta, Canada

[5]Bruyère Research Institute, Ottawa, Ontario, Canada

CORRESPONDING AUTHOR: Monique Potvin Kent (monique.potvinkent@uottawa.ca)

Survey for parents – Part A only

Domain	Survey Question
**Province of residence**	1. What province do you currently live? [SINGLE SELECT]<br>a) Alberta<br>b) British Columbia<br>c) Manitoba<br>d) New Brunswick<br>e) Newfoundland<br>f) Nova Scotia<br>g) Ontario<br>h) Prince Edward Island<br>i) Quebec<br>j) Saskatchewan<br>k) I do not live in Canada<br>*[TERMINATE IF NOT ONTARIO OR QUEBEC]*
**Children in household**	2. Are you the parent/guardian of any children under the age of 18 living in your household? *[SINGLE SELECT]*<br>a) Yes<br>b) No *[TERMINATE]*
**Age and sex of children in the household**	*3.* Please indicate the age and sex assigned at birth of any children under the age of 18 living in your household. Please select all that apply. *[MULTISELECT GRID]*<br>*[ROWS]*<br>a) Age 1 or younger<br>b) Age 2<br>c) Age 3<br>d) Age 4<br>e) Age 5<br>f) Age 6<br>g) Age 7

Domain	Survey Question
	h) Age 8<br>i) Age 9<br>j) Age 10<br>k) Age 11<br>l) Age 12<br>m) Age 13<br>n) Age 14<br>o) Age 15<br>p) Age 16<br>q) Age 17<br><br>*[COLUMNS]*<br>Male<br>Female<br><br>*PROGRAMMER NOTE: If respondent does NOT have any children aged 10-17 years TERMINATE*
**Parent/Guardian pre-screening**	4. *UNIVERSE: Respondents who indicated they had a child between the ages of 10-17 years living in the household.*<br>You indicated that you have a child (or children) between the ages of 10 and 17 years living in your household.<br>We would like you to answer a few more questions and have your child complete a survey if they qualify.<br>Please indicate below if you are willing to let your child participate? Their participation will take about 12-15 minutes.<br>Please note that your child does not need to be available right now to participate. After you complete the questions for the parent/guardian section, instructions will be provided for your child's participation. *[SINGLE SELECT]*<br>Yes → *[REDIRECT PANELIST TO SURVEY]*<br>No → Thank you for your time. *[TERMINATE]*

Domain	Survey Question
**Parent/Guardian consent**	5. We would like to conduct a survey with ONE of your CHILDREN AGED 10-17 years. Please read the following information and indicate if you are willing to let your child participate.<br><br>- My child and I are invited to participate in a research survey conducted by Dr. Monique Potvin Kent at the University of Ottawa. This study is funded by the Canadian Institutes of Health Research and has been approved by the University of Ottawa Research Ethics Board (**H-11-21-7343**).<br>- The purpose of this survey is to see whether the amount and types of advertisements seen by children and teens online and on social media differs by gender. The purpose of this study is also to see how children and teens interact with promotional messages online.<br>- My participation consists of answering a few questions on my socio-economic status (e.g., income, education, etc.). **Completing these questions will take about 8-10 minutes.**<br>- My child will also be asked to complete a consent form.<br>- **The participation of my child will include completing one 12 to 15-minute survey on their demographics (age, sex at birth, and gender), social media usage, and exposure to food advertisements.**<br>- This survey will be administered and submitted through Leger.<br>- My participation and that of my child will provide valuable information on food and beverage advertising to children and teens on digital media. This information may inform policy restricting digital food and beverage marketing to youth in Canada.<br>- The information that my child and I provide will only be used for the study. Our anonymity will be protected in the following manner**:** Information regarding our identities such as name and contact information will be assigned a unique participant number. The document linking our identities and this number will be kept separately from our survey responses and will only be accessible to the Principal Researcher and her research team. Our names will not be included in any written reports and our identity will not be revealed in any situation.<br>- The research team will implement security safeguards to protect all survey data, however there is still a risk your child's responses may be intercepted by a third-party (e.g., hacker).<br>- Data collected will be kept on a secured computer and will be protected with a password. The only people who will have access to the research data are the Principal Researcher named above and her research team. The data will be kept for indefinitely after the end of the project.

Domain	Survey Question
	- I will be compensated by the panel according to their usual incentive structure. We encourage you to share this incentive with your child.<br>- I understand that my child and I are under no obligation to participate and if we choose to participate, we can withdraw from the study at any time and/or refuse to answer any questions, without suffering any negative consequences. If we choose to withdraw, all data gathered until the time of withdrawal will be destroyed.<br>- If I have any questions regarding the ethical conduct of this study, I may contact the Protocol Officer for Ethics in Research, University of Ottawa, Tabaret Hall, 550 Cumberland Street, Room 154, Ottawa ON K1N 6N5 Tel.: (613) 562-5387 Email: ethics@uottawa.ca<br>- For all other questions about the study, please contact the Principal Researcher, Dr. Monique Potvin at mpotvink@uottawa.ca, or her Research Coordinator, Carolina Soto at csoto007@uottawa.ca.<br>*Please note that your child does not need to be available right now to participate. After you complete the questions for parents/guardians, instructions will be provided for having your child participate when they are available.*<br><br>**Would you be willing to allow your child to participate in this survey?** *[SINGLE SELECT]*<br>By clicking "Yes" below, you confirm that you are the parent/legal guardian of the child participating in this study and agree to allow them to participate.<br><br>**Yes →** *[Continue]*<br>**No →** *[TERMINATE]*
**Parental Status**	6. What is your relationship with the child participating in the study? *[SINGLE SELECT]*<br>a. Mother<br>b. Father<br>c. Other, specify: ______________________
**Perceived income adequacy**	7. Thinking about your total monthly income, how difficult or easy is it for you to make ends meet? *[SINGLE SELECT]*<br>a) Very difficult<br>b) Difficult

Domain	Survey Question
	c) Neither easy nor difficult<br>d) Easy<br>e) Very easy<br>f) Prefer not to answer
**Household income**<br>Adapted from:<br>**https://www.publichealthontario.ca/-/media/documents/nCoV/main/2020/06/introducing-race-income-household-size-language-data-collection**	8. What is your best estimate of your total household income received by all household members, from all sources, before taxes and deductions, during the year ending December 31, 2022? Select one from the list of income ranges: *[SINGLE SELECT]*<br>a) 0 - $29,999<br>b) $30,000-$49,999<br>c) $50,000-$69,999<br>d) $70,000-$99,999<br>e) $100,000-149,999<br>f) $150,000 or more<br>g) Prefer not to answer<br><br>9. Including yourself, how many family members live in your household? *[OE FOR DIGITS ONLY AND PNTA BUTTON]*<br>_________(people)<br>Prefer not to answer
**Age of participating child**	10. How old is your participating child? QUOTAS FOR 10-12. *[SINGLE SELECT]*<br>a. Younger than 10 years<br>b. 10 years<br>c. 11 years<br>d. 12 years<br>e. 13 years<br>f. 14 years<br>g. 15 years<br>h. 16 years<br>i. 17 years<br>j. 18 years or older

Domain	Survey Question
	[Terminate survey if <10 or >17 years]
**Child sex**	11. What was ~~your~~ this child's sex at birth? *[SINGLE SELECT]*<br>a) Male<br>b) Female
**Race of child**<br>https://www.cihi.ca/sites/default/files/document/guidance-and-standards-for-race-based-and-indigenous-identity-data-en.pdf	12. In our society, people are often described by their race or racial background. These are not based in science, but our race may influence the way we are treated by individuals and institutions, and this may affect our health.<br>Which category(ies) best describes your child? Check all that apply: *[MULTISELECT]*<br><br>a) Black (e.g. African, Afro-Caribbean, African Canadian)<br>b) East Asian (e.g. Chinese, Korean, Japanese, Taiwanese descent)<br>c) South Asian (e.g. Indian, Pakistani, Bangladeshi, Sri Lankan, Indo-Caribbean)<br>d) Southeast Asian (e.g. Cambodian, Filipino, Indonesian, Thai, Vietnamese, or other Southeast Asian descent)<br>e) Indigenous (e.g. First Nations, Métis, Inuk/Inuit)<br>f) Latin American (e.g. Latin American, Hispanic)<br>g) Middle Eastern (e.g. Arab, Persian, West Asian descent including Afghan, Egyptian, Iranian, Lebanese, Turkish, Kurdish)<br>h) White (e.g. European descent)<br>i) Another race category (please specify): _______<br>j) Prefer not to answer *[EXCLUSIVE]*
**QFILT#1**	
**Language**<br>https://www.cihi.ca/sites/default/files/document/guidance-and-standards-for-race-based-and-indigenous-identity-data-en.pdf	*13.* What language(s) does this child speak on a regular basis at home? *[MULTISELECT]*<br>a) English<br>b) French<br>c) Other language(s) — specify: *[open text]*<br><br>*Programer: If more than one selection for Q13*

Domain	Survey Question
	14. Of these languages, which one does this child speak most often at home? Indicate more than one language only if they are spoken equally at home. *[MULTISELECT]*<br>a) English<br>b) French<br>c) Other language(s) — specify: *[open text]*
**Parent-reported height**	15. How tall is the participating child without shoes? Would you rather answer in:<br>a. Feet and inches<br>b. Centimetres<br>c. Prefer not to answer [*Send panelist to Q17*]<br><br>Enter feet: _______ ft AND enter inches: ______ in<br>Enter centimetres: _____ cm
**Height source**	16. How did you obtain the participating child's height? *[SINGLE SELECT]*<br>a. My child told me their height<br>b. I estimated or guessed<br>c. I used a tape measure or yardstick within the past six months<br>d. My child was measured at the doctor's office within the past six months<br>e. I determined their height in some other way
**Parent-reported weight**	17. How much does your child weigh without clothes or shoes? Would you rather answer in:<br>a. Pounds (lb)<br>b. Kilograms (kg)<br>c. Prefer not to answer [*Send panelist to Q19*]<br><br>Enter pounds: ______ lb<br>Enter kilograms: ______ kg
**Weight source**	18. How did you obtain your child's weight? *[SINGLE SELECT]*<br>a. My child told me their weight<br>b. I estimated or guessed

Domain	Survey Question
	c. I used a scale within the past six months<br>d. My child was weighed at the doctor's office within the past six months<br>e. I determined their weight in some other way
**Handover to child**	19. The remaining questions should be completed by your child.<br><br>If your child is not available right now, they may complete the survey later.<br><br>Please close the survey and come back when your child is available. If you have received an email invite, you can click the original survey link from that email and your child will be able to continue where you left off. If you have yet to receive an email, you can access again through your LEO Account.

Survey for children (Part A)

Domain	Survey Question
**Child assent**	20. Before you start, please read below and let us know if you agree to participate.<br><br>- I am invited to participate in this research survey. The person in charge of this study is Dr. Monique Potvin Kent at the University of Ottawa.<br>- The goal of this study is to see if there are differences between boys, girls and children of other genders in the types and number of food and beverage advertisements that they see on social media.<br>- **My participation consists of filling out an online survey on my media use, food habits, and whether I have seen any food and beverage ads online. The survey will take about 12-15 minutes.**<br>- My participation will provide valuable information on how food and beverage advertising is viewed by children and teens online.

Domain	Survey Question
	- The information that I provide will only be used for this study and will only be accessible to the researcher and her team. My privacy will be respected. The research team will not tell anybody else that I have been part of this study. In order to protect my privacy, my name will be assigned a unique participant number. The document linking my identity and this number will be kept separate from my survey responses and will only be accessible to the Principal Researcher and her team. My name will not be included in any written reports and my identity will not be revealed in any situation.<br>- My parent will receive financial compensation (money) for our participation in this study.<br>- I am not required to participate in this research project. If I choose to participate, I can refuse to answer one or more questions or I can stop participating without anything bad happening to me or my parent/guardian (I will not be punished).<br>- If I have any questions regarding the ethics of this study, I may contact the Protocol Officer for Ethics in Research, University of Ottawa, Tabaret Hall, 550 Cumberland Street, Room 154, Ottawa ON K1N 6N5 Tel.: (613) 562-5387 Email: ethics@uottawa.ca<br>- For all other questions about the study, please contact Dr. Monique Potvin Kent at mpotvink@uottawa.ca, or her Research Coordinator, Carolina Soto at csoto007@uottawa.ca.<br><br>Do you agree to participate in this survey? *[SINGLE SELECT]*<br><br>**Yes** → *[CONTINUE]*<br>**No** → ~~Thank you for your time. You will now be redirected back to the survey company.~~ [TERMINATE]
**Gender**	21. What gender do you identify as? (Gender refers to who you know yourself to be, which may be different from your sex assigned at birth and may be different from what is indicated on legal documents.) *[SINGLE SELECT]*<br><br>Is it:<br>a) Boy<br>b) Girl<br>c) I identify as ____________ (please specify) *[OE]*
**Ownership of electronic device**	22. Do you have your own…: *[SINGLE SELECT GRID]*

Domain	Survey Question
	Smartphone (iPhone, Android, Google phone, Pixel, etc.)<br>Tablet (iPad, Samsung tablet, etc.)<br>Laptop or desktop computer<br><br>*[PROGRAMMER NOTE: Show options for each as radio buttons]*<br>a) Yes<br>b) No, but I use a family device<br>c) No, and I do not use a family device<br>d) Prefer not to answer
**Usual digital media use**	How long do you usually spend using a digital device (smartphone, tablet, laptop or desktop computer)…<br>23. when you're **at school or doing homework** …<br>…. on a typical weekday? _____hr _____min<br>…. on a typical weekend day? _____hr _____min<br>Prefer not to answer<br><br>24. when you're **NOT** at school or doing homework …<br>…. on a typical weekday? _____hr _____min<br>…. on a typical weekend day? _____hr _____min<br>Prefer not to answer
	25. Which social media platforms (if any) do you use? *[MULTISELECT]*<br><br>*Select all that apply.*<br>Instagram<br>YouTube<br>TikTok<br>Facebook (including Facebook Messenger)<br>Twitter<br>Snapchat<br>Reddit

Domain	Survey Question
	Pinterest<br>Twitch<br>Discord<br>Other: Please specify: __ *[OE]*<br>Prefer not to answer<br>*[PROGRAMMER NOTE: If respondent selects* "Prefer not to answer"*, skip to Q28]*<br><br>26. Please rank the social media platforms you selected in order from <u>most used to least used</u>:<br>*[PROGRAMMER NOTE: Populate options selected on Q26. Allow respondent to drag items into their preferred order using an interactive list]*<br><br>27. What are the <u>5 websites that you visit most often</u> in your free time when you are not doing schoolwork? Please do NOT include search engines like Google or social media platforms like Facebook or Instagram.<br>____<br>____<br>____<br>____<br>____<br>Prefer not to answer
	28. On a normal **weekday**, how much time do you spend: *[SINGLE SELECT GRID]*<br>Watching YouTube<br>On social media (including messaging, posting, or liking posts on platforms such as Instagram, Twitter, TikTok, Snapchat, Facebook)<br>Watching TV shows, series, or movies on television streaming platforms (like Disney+, Prime Video, etc.)<br>Playing games on smartphones, computers, or game consoles<br>Browsing online, reading websites, Googling, etc.<br>Watching gaming or livestreaming content on Twitch<br><br>*[PROGRAMMER NOTE: Show options for each as radio buttons]*<br>a) 0 hours (none)

Domain	Survey Question
	b) Up to 15 minutes<br>c) Up to 30 minutes<br>d) Up to 1 hour<br>e) Up to 2 hours<br>f) Up to 3 hours<br>g) Up to 4 hours<br>h) More than 4 hours<br>i) Prefer not to answer<br><br>29. On a normal **weekend**, how much time do you spend: *[SINGLE SELECT GRID]*<br>Watching YouTube<br>On social media (including messaging, posting, or liking posts on platforms such as Instagram, Twitter, TikTok, Snapchat, Facebook)<br>Watching TV shows, series, or movies on television streaming platforms (like Disney+, Prime Video, etc.)<br>Playing games on smartphones, computers, or game consoles<br>Browsing online, reading websites, Googling, etc.<br>Watching gaming or livestreaming content on Twitch<br><br>*[PROGRAMMER NOTE: Show options for each as radio buttons]*<br>j) 0 hours (none)<br>k) Up to 15 minutes<br>l) Up to 30 minutes<br>m) Up to 1 hour<br>n) Up to 2 hours<br>o) Up to 3 hours<br>p) Up to 4 hours<br>q) More than 4 hours<br>r) Prefer not to answer
**QFILT#2**	

Domain	Survey Question
**Self-reported exposure to unhealthy food marketing – frequency and type**	30. Think about the last 7 days. **How often** did you see or hear advertisements for the food or drinks listed below while using your smartphone, tablet, laptop or desktop computer? *[SINGLE SELECT GRID]*<br>Ads for sugary drinks (soda/pop, sports drinks, energy drinks, juices, etc.)<br>Ads for sugary cereals<br>Ads for fruit or vegetables<br>Ads for salty/savoury snacks like chips, pretzels, cheese puffs, etc.<br>Ads for fast foods like pizza, French fries, or burgers<br>Ads for desserts or treats like cookies, ice cream, or candy<br>Ads for alcohol like vodka, rum, beer, wine, coolers, or shots<br><br>[*Show options for each as radio buttons*]<br>a) Never<br>b) 1-3 times during the week<br>c) 4-6 times during the week<br>d) Every day<br>e) More than once a day<br>f) Prefer not to answer
**Self-reported exposure to unhealthy food advertising (location)**	31. In the past 7 days, **where** did you see or hear advertisements for 'unhealthy' foods or drinks? *Unhealthy food and drinks include packaged foods high in sugar, salt, or fats, such as soda/pop, fast food, chips, sugary cereals, cookies, and chocolate bars. [SINGLE SELECT GRID]*<br>Facebook<br>Instagram<br>Twitter<br>TikTok<br>Snapchat<br>Twitch<br>YouTube<br>Pinterest<br>Gaming websites (e.g., Roblox)<br>Television streaming platforms (e.g., Netflix, Crave, Prime Video, Disney+)<br>Blogs/websites

Domain	Survey Question
	Spotify<br>Livestreamed gaming or eSports<br>Posts and videos shared by influencers<br>Posts and videos shared by friends in social media<br>a. Yes<br>b. No<br>c. I don’t use this platform<br>d. Prefer not to answer<br><br>*[Y/N options shown for each marketing location]*
**Self-reported exposure to digital marketing techniques (Part 1 – General strategies)**	32. In the past 7 days, have you seen unhealthy food or drinks advertised while using your smartphone, tablet, laptop, or desktop computer, with any of the following? *[SINGLE SELECT GRID]*<br>Actors who are kids or teenagers<br>Slang used by kids or teenagers<br>Appealing bright colours<br>Themes such as magic, mystery, adventure, or heroes<br>Themes like fun, popularity, being cool, being fashionable, being free and independent<br>Extreme sports<br>New products<br>Reward/incentive programs (collecting points or rewards that can be used to purchase food/beverages)<br>Contests<br>Free giveaways<br>Price promotions (buy 2 for the price of 1)<br>Limited time offers (offers that are only good up until a certain date)<br>Athletes, favourite sports teams<br>Celebrities from movies, TV, or bands (pop, rock, rap stars)<br>Social media influencers (like Mr. Beast or Charli D’amelio)<br>Characters owned by food companies (like Tony the Tiger or Snap, Crackle, and Pop)<br>Cartoon characters from movies or TV (like Bart Simpson or Spiderman)<br>Special effects/animation<br>Activities, polls, quizzes, or games<br>Catchy/popular music

Domain	Survey Question
	References to health or nutrition (like "This is part of a healthy breakfast!")<br>Saying the product is convenient<br>Link to an event (like the FIFA World Cup or a music festival)<br>Link to a movie or TV show<br>Encouragement to like, comment, or share with your friends on social media<br>a) Yes<br>b) No<br>c) Prefer not to answer<br><br>*[Options asked for each technique]*
**Self-reported exposure to digital marketing techniques (Part 2: social media influencers)**	33. Do you have any favourite social media influencers or internet celebrities? *[SINGLE SELECT]*<br>a. Yes<br>b. No [*Send panelist to Q37*]<br>c. Prefer not to answer [*Send panelist to Q37*]<br><br>*Universe: Respondents who answered 'Yes' to Q34*<br><br>34. If yes, please list your top 3 favourite social media influencers *[3 OE BOXES]*<br>__________<br>__________<br>__________<br><br>35. In the past 7 days, have you seen any social media influencers mention unhealthy food or drinks or include these products in their posts? *[SINGLE SELECT]*<br>a. Yes<br>b. No<br>c. Prefer not to answer
**Impact of marketing techniques – purchase**	36. Have you ever bought food/beverage products because you saw them advertised online? *[SINGLE SELECT]*<br>a. Yes<br>b. No

Domain	Survey Question
	c. Prefer not to answer
	37. Have you ever asked your parents to buy food/beverage products because you saw them advertised online? *[SINGLE SELECT]*<br>a. Yes<br>b. No<br>c. Prefer not to answer
**Gender-based advertisements**	38. In advertisements that feature people or fictional characters, more often you see: *[SINGLE SELECT]*<br>a. Boy characters only<br>b. Girl characters only<br>c. Other genders<br>d. Both boy and girl characters<br>e. Both boy, girl and other genders<br>f. Prefer not to answer<br><br>39. If you were to see a food and beverage ad, would you be more interested in the product if: *[SINGLE SELECT]*<br>a) A boy is advertising the product<br>b) A girl is advertising the product<br>c) Other genders are advertising the product<br>d) It doesn't matter to me<br>e) Prefer not to answer
**Food companies - engagement**	40. Have you ever liked, shared, or followed any food or beverage companies (e.g., McDonald's, Coca-Cola, or restaurants) on social media? *[SINGLE SELECT]*<br>a. Yes<br>b. No [*Send panelist to Q44*]<br>c. Prefer not to answer [*Send panelist to Q44*]<br><br>*Universe: Respondents who answered 'Yes' to Q41*

Domain	Survey Question
	41. If yes, which ones: *[3 OE BOXES]*<br><br>__________<br>__________<br>___________
	42. *Universe: Respondents who answered 'Yes' to Q41*<br>Why do you follow restaurant, food and/or beverage companies on social media? (Select all that apply) [*Programmer please randomize order*.*] [MULTISELECT]*<br>a. I get deals on products<br>b. I like to find out about new products<br>c. The posts are funny<br>d. The posts are interesting<br>e. The posts that show food and beverages look really good/tasty/delicious<br>f. The posts tell me about special events in my city or town<br>g. I can win contests or prizes<br>h. I can donate to worthy causes<br>i. I like the posts<br>j. I like to share these posts with my friends<br>k. Other. Please specify: __*[OE]*
**Food companies – mobile apps**	43. Do you have any apps for restaurants or food/beverage companies (for example, the McDonald's or RedBull TV app) on your smartphone? *Here, we do NOT mean food delivery service apps such as UberEats, Skip the Dishes, etc. [SINGLE SELECT]*<br>a. Yes<br>b. No [*Send panelist to Q46*]<br>c. Prefer not to answer [*Send panelist to Q46*]<br><br>*Universe: Respondents who answered 'Yes' to Q44.*<br>44. Please list the restaurant and food company apps that you have on your smartphone: [*open text*]

Domain	Survey Question
**Food delivery service apps**	45. Do you have any food delivery service apps (like UberEats, Skip the Dishes) on your smartphone? *[SINGLE SELECT]*<br>a. Yes<br>b. No [*Send panelist to Q48*]<br>c. Prefer not to answer [*Send panelist to Q48*]
	Universe: Respondents who answered 'Yes' to Q46.<br>46. Which food delivery apps do you have on your smartphone? *Select all that apply*. *[MULTISELECT]*<br>a. UberEats<br>b. SkipTheDishes<br>c. GrubHub<br>d. DoorDash<br>e. Foodora<br>f. Other. Please specify: ____________ *[OE]*<br>g. Prefer not to answer *[EXCLUSIVE]*
**Self-reported exposure to digital marketing techniques (Part 3: Other techniques)**	47. In the past 7 days, have you received any pop-up notices on your phone from apps that include unhealthy food and beverage advertisements? *Reminder: Unhealthy food and drinks include packaged foods high in sugar, salt, or fats, such as soda/pop, fast food, chips, sugary cereals, cookies, and chocolate bars. [SINGLE SELECT]*<br>a. Yes<br>b. No<br>c. Prefer not to answer
	48. Think about the last 7 days. how many days did you have a meal (breakfast, lunch, dinner, or snack) from a sit-down restaurant, fast food restaurant or food stand outside of school? *[SINGLE SELECT]*<br>a. 0 days (not at all)<br>b. 1 day<br>c. 2 days<br>d. 3 days<br>e. 4 days

Domain	Survey Question
**Dietary behaviours**	f. 5 days<br>g. 6 days<br>h. 7 days (every day)<br>i. Prefer not to answer
	49. Think about the last 7 days. How many days did you have any sugary drinks (soda/pop, sports drinks, energy drinks, juices etc.)? *[SINGLE SELECT]*<br>a) 0 days (not at all)<br>b) 1 day<br>c) 2 days<br>d) 3 days<br>e) 4 days<br>f) 5 days<br>g) 6 days<br>h) 7 days (every day)<br>i) Prefer not to answer
	50. Think about the last 7 days. How many days did you have any salty/savoury snacks (chips, pretzels, cheese puffs, etc.)? *[SINGLE SELECT]*<br>a) 0 days (not at all)<br>b) 1 day<br>c) 2 days<br>d) 3 days<br>e) 4 days<br>f) 5 days<br>g) 6 days<br>h) 7 days (every day)<br>i) Prefer not to answer
	51. Think about the last 7 days. How many days did you have any desserts or sweet treats (ice cream, cake, cookies, candy, chocolate etc.)? *[SINGLE SELECT]*<br>a) 0 days (not at all)

Domain	Survey Question
	b) 1 day<br>c) 2 days<br>d) 3 days<br>e) 4 days<br>f) 5 days<br>g) 6 days<br>h) 7 days (every day)<br>i) Prefer not to answer
**QFILT#3**	
**Perceived target audience**	*52.* Please pick who you think advertising for this product is usually meant for …<br>[*show image and product name*] *[SINGLE SELECT GRID]*<br><br>McDonald's Big Mac<br>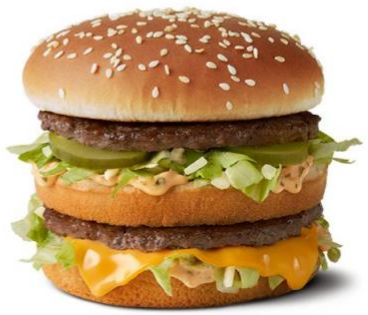<br>McDonald's fries

Domain	Survey Question
	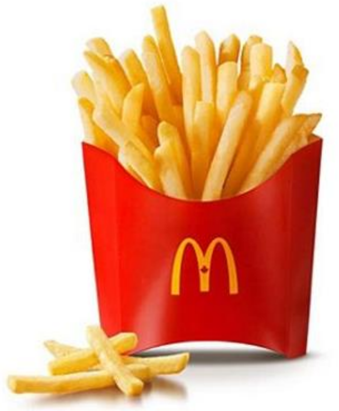<br>Starbucks Frappuccino<br>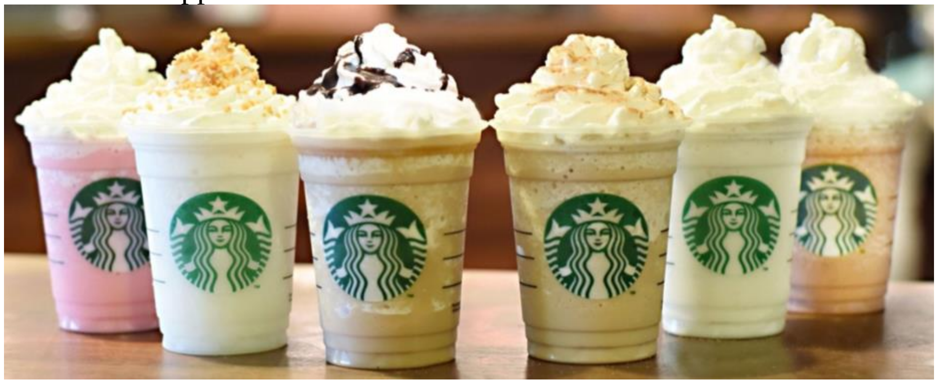<br><br>Pepsi cola

Domain	Survey Question
	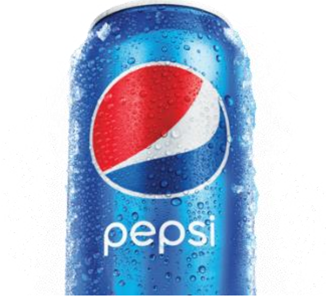<br><br>Red Bull<br>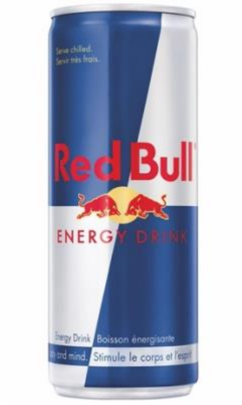<br><br>Doritos

Domain	Survey Question
	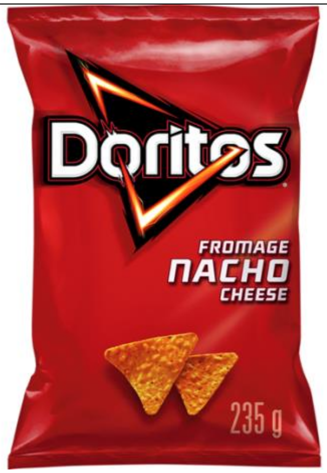<br><br>Skittles<br>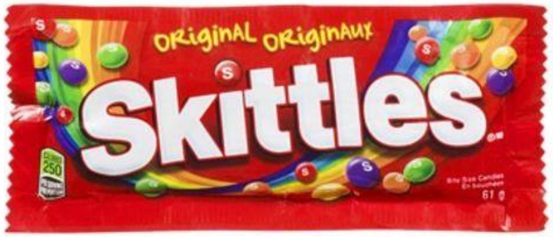<br><br>Nutella

Domain	Survey Question
	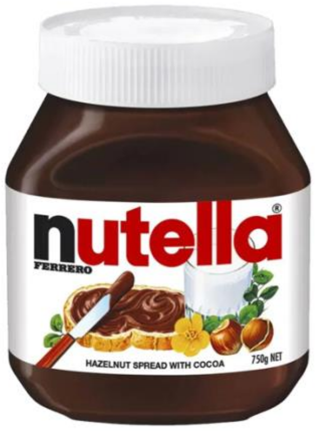<br><br>*[PROGRAMMER NOTE: Show options for each as radio buttons]*<br>a. Boys<br>b. Girls<br>c. Other genders<br>d. Both boys and girls<br>e. Both boys, girls and other genders<br>f. I've never seen an advertisement for this brand or product<br>g. Prefer not to answer
**Self-reported exposure to alcohol by setting/media channel**	53. **Think about the last 7 days**.<br>Have you seen or heard advertisements for **alcohol** in any of these places?<br>*Alcohol includes beer, wine, cider, liquor/spirits (e.g., rum, gin, vodka), mixed drinks or cocktails, spritzers, alcopops/coolers and any other beverage containing alcohol.*<br><br>*Select all that apply. [MULTISELECT]*<br>TV shows, series or movies<br>Websites<br>Social media

Domain	Survey Question
	Video or computer games<br>Restaurants (e.g. on signs, tray linings, coasters, awnings, menu boards)<br>Food delivery apps<br>Stores (such as posters, product displays)<br>Radio<br>Magazines or newspapers<br>Billboards<br>Buses, bus stops and other public transit<br>Movie theatres<br>School<br>Recreation or community centres<br>At a sports event, concert, or community event<br>During professional sports games or events watched on television or online<br>Contests, free samples, or coupons<br>Other (please specify): [*open-ended*]<br>I haven't seen any ads for alcohol in the last 7 days *[EXCLUSIVE]*<br>Prefer not to answer *[EXCLUSIVE]*
**Self-reported exposure in social media**	54. In the last 7 days, have you seen or heard advertisements for **alcohol** online in any of these places? *[SINGLE SELECT GRID]*<br>Facebook<br>Instagram<br>Twitter<br>TikTok<br>Snapchat<br>Twitch<br>Snapchat<br>YouTube<br>Pinterest<br>Spotify<br>Discord<br>Livestreamed gaming or eSports

Domain	Survey Question
	Posts and videos shared by celebrities or influencers<br>Posts and videos shared by friends in social media<br><br>*[PROGRAMMER NOTE: Show options for each as radio buttons]*<br><br>a) Yes<br>b) No<br>c) Prefer not to answer
**Top of mind recall / brand awareness**	55. An example of a candy brand is Skittles. An example of a beverage brand is Coca Cola.<br><br>Please name up to 5 **alcohol** brands:<br>Brand 1: [open-text]<br>Brand 2: [open-text]<br>Brand 3: [open-text]<br>Brand 4: [open-text]<br>Brand 5: [open-text]<br>I don't know any alcohol brands *[EXCLUSIVE]*<br>Prefer not to answer *[EXCLUSIVE]*
**Alcohol consumption**<br><br>**Reference:** **https://www.info-cstads.ca/en/Survey**	56. In the **last 30 days,** how often did you have a drink of alcohol that was more than just a sip? *[SINGLE SELECT]*<br>a) I have never had a drink of alcohol that was more than just a sip<br>b) I have not done this in the last 30 days<br>c) Once or twice<br>d) Once or twice a week<br>e) 3 or 4 times a week<br>f) 5 or 6 times a week<br>g) Every day<br>h) Prefer not to answer
